# Butyrate Protects Rat Liver against Total Hepatic Ischemia Reperfusion Injury with Bowel Congestion

**DOI:** 10.1371/journal.pone.0106184

**Published:** 2014-08-29

**Authors:** Bin Liu, Jianmin Qian, Qingbao Wang, Fangrui Wang, Zhenyu Ma, Yingli Qiao

**Affiliations:** 1 Department of General Surgery, Huashan Hospital, Fudan University, Shanghai, China; 2 Department of General Surgery, Affiliated Hospital of Taishan Medical College, Taian, Shandong, China; 3 Department of General Surgery, Liaocheng People's Hospital, Affiliated Liaocheng Hospital of Taishan Medical College, Liaocheng, Shandong, China; University Hospital Heidelberg, Germany

## Abstract

Hepatic ischemia/reperfusion (I/R) injury is an unavoidable consequence of major liver surgery, especially in liver transplantation with bowel congestion, during which endotoxemia is often evident. The inflammatory response aggravated by endotoxin after I/R contributes to liver dysfunction and failure. The purpose of the present study was to investigate the protective effect of butyrate, a naturally occurring four-carbon fatty acid in the body and a dietary component of foods such as cheese and butter, on hepatic injury complicated by enterogenous endotoxin, as well as to examine the underlying mechanisms involved. SD rats were subjected to a total hepatic ischemia for 30 min after pretreatment with either vehicle or butyrate, followed by 6 h and 24 h of reperfusion. Butyrate preconditioning markedly improved hepatic function and histology, as indicated by reduced transaminase levels and ameliorated tissue pathological changes. The inflammatory factors levels, macrophages activation, TLR4 expression, and neutrophil infiltration in live were attenuated by butyrate. Butyrate also maintained the intestinal barrier structures, reversed the aberrant expression of ZO-1, and decreased the endotoxin translocation. We conclude that butyrate inhibition of endotoxin translocation, macrophages activation, inflammatory factors production, and neutrophil infiltration is involved in the alleviation of total hepatic I/R liver injury in rats. This suggests that butyrate should potentially be utilized in liver transplantation.

## Introduction

Hepatic ischemia/reperfusion (I/R) injury remains a major complication of major liver surgery, including liver transplantation and hepatectomy [1]. I/R resulting in Kupffer cell/neutrophil activation and cytokine release often leads to liver dysfunction, and even acute and chronic rejection after transplantation, especially when grafts from non-heart-beating donors are used [2], [3]. Moreover, portal triad clamping interrupts the flow of mesenteric blood, resulting in intestinal ischemia, congestion, and damage to the intestinal barriers, thereby accelerating bacterial translocation and intestinal endotoxemia, which complicates I/R-induced injury [4], [5], [6]. It has been demonstrated that endotoxin can aggravate I/R-induced liver injury by oxidative stress, free radical formation, and the release of inflammatory mediators [7]. Therefore, it is worthwhile to investigate novel agents that can protect against total hepatic I/R injury, especially that aggravated by endotoxemia.

Butyrate, a four-carbon short-chain fatty acid (SCFA) derivative found in foods such as parmesan cheese and butter, which is also normally produced by bacterial fermentation of unabsorbed carbohydrates, has received considerable attention as a potential therapeutic agent for cancers because of its histone deacetylase (HDAC) inhibition activity [8]. In addition to the anticancer activity of SCFAs, recent data have demonstrated that they have potent anti-inflammatory or immunomodulatory and anti-oxidant effects at non-cytotoxic dosing levels [9]. Moreover, SCFAs are the favored energy source for colonic epithelial cells and are important for normal intestinal biology [10]. Furthermore, in physiological concentrations, SCFAs-and especially butyrate-can establish and maintain the intestinal mucosal barrier, as shown in previous in vitro studies [11], [12].

We have demonstrated that the dose of 300 mg/kg of butyrate exhibits anti-inflammatory and hepatoprotective effects in a rat model of partial hepatic ischemic reperfusion [13]. However, its effect on total hepatic injury complicated by endotoxin translocation from the intestinal lumen in the setting of liver transplantation has not been previously evaluated. Since impaired intestinal barriers may further promote endotoxin release, thereby aggravating damage to liver tissues induced by I/R, and butyrate can enhance intestinal barrier function, we hypothesize that butyrate may also ameliorate total hepatic injury through the inhibition of endotoxin gut leakage.

## Materials and Methods

### Animals

Male Sprague-Dawley rats (200–250 g) were housed in the Department of Laboratory Animal Science at Fudan University with a laminar flow, specific pathogen-free atmosphere. Animal protocols were approved by the Fudan University Animal Care Committee, and the experiments were performed in adherence to the guidelines provided by the National Institutes of Health for the use of animals in laboratory experiments.

### Total hepatic warm I/R

Total hepatic warm ischemia was induced as previously described [14]. All surgical procedures were carried out under sterile conditions. In brief, rats were laparotomized and a sterile pediatric vessel loop was placed around the portal triad for 30 min to induce total hepatic ischemia and mesenteric congestion. Sham controls underwent the same procedure without vascular occlusion. Reperfusion was initiated by removal of the loop. The rectal temperature was maintained at 37°C throughout surgery by a warming pad. For the pretreatment experiments, some rats were injected intravenously with 300 mg/kg of sodium butyrate (Sigma, Saint Louis, USA), as we previously described [13], or vehicle (normal saline solution) at 30 min prior to ischemia.

### Serum aminotransferase assessment

To assess hepatic function and cellular injury following total liver ischemia, serum aspartate aminotransferase (AST) and alanine aminotransferase (ALT) activities were measured in blood samples obtained at predetermined time points (6 and 24 h) after reperfusion with a standard automatic analyzer (type 7150; Hitachi, Tokyo, Japan).

### Histological examination

Liver tissues and the last 10 cm of the ileum were harvested, fixed by immersion in 4% buffered paraformaldehyde, and embedded in paraffin. Sections (4 µm) were stained with hematoxylin-eosin (HE) and assessed for inflammation and tissue damage.

### Liver myeloperoxidase activity

Tissue-associated myeloperoxidase (MPO) activity, an indicator of neutrophil infiltration, was determined as previously described [15].

### Real-time reverse-transcriptase polymerase chain reaction

Total RNA was extracted from the liver using TRIzol reagent (Life Technologies, Carlbad, USA) according to the manufacturer’s instructions. The mRNA for tumor necrosis factor-alpha (TNF-a), interleukin-6 (IL-6), and glyceraldehyde 3-phosphate dehydrogenase (GAPDH) was quantified in duplicate by SYBR green two-step, real-time reverse-transcriptase polymerase chain reaction (RT-PCR) with an ABI-Prism 7500 Sequence Detector (Applied Biosystems, Foster City, USA) using the primers previously described [15]. GAPDH mRNA levels were used as the invariant control for each sample.

### Enzyme-linked immunosorbent assay

TNF-α, IL-6, and endotoxin levels in serum were measured using enzyme-linked immunosorbent assay (ELISA) kits (R&D Systems, Minneapolis, USA).

### Immunohistochemistry

Anti-ZO-1 antibody (Abcam, Cambridge, UK) was used for immunohistochemical staining. Immunohistochemical detection was performed using a two-step visualization system (DAKO, Glostrup, Denmark).

### Immunofluorescence

Anti-CD68 antibody (AbD Serotec, Kidlington, UK) was used for immunofluorescent staining. Secondary antibody was FITC-conjugated IgG antibody (Santa Cruz Biotechnology, Dallas, USA), and the nuclei were labeled with DAPI (Invitrogen, Camarillo, CA).

### Western blot

The methodology of Western blot analysis has been described previously [15]. Primary antibodies were monoclonal antibody to ZO-1, TLR 4, GAPDH (Abcam) and CD68 (AbD Serotec), the secondary antibody was HRP-conjugated IgG antibody (Santa Cruz Biotechnology, Dallas, USA). Proteins were visualized by an enhanced chemiluminescence assay kit (GE Healthcare, Buckinghamshire, UK) and the levels of proteins were normalized with respect to GAPDH band density.

### Transmission electron microscopy

Ultrathin sections of ileal segments were prepared using standard techniques and examined using a JEM 1200-EX transmission electron microscope (Hitachi, Tokyo, Japan).

### TUNEL staining

TUNEL staining was performed on paraffin sections using an in situ cell death detection kit (Roche, Basel, Switzerland), and the nuclei were labeled with DAPI (Invitrogen). The number of TUNEL positive cells was counted under a microscope (×200) in five fields followed by averaging.

### Statistical analysis

Group sizes are indicated in the figure legends. Data are presented as means ± SD, unless otherwise noted. Statistical analysis was performed via either an ANOVA or Kaplan-Meier test. A difference of p<0.05 was considered statistically significant.

## Results

### Butyrate attenuates total hepatic I/R injury

**Figure 1:**
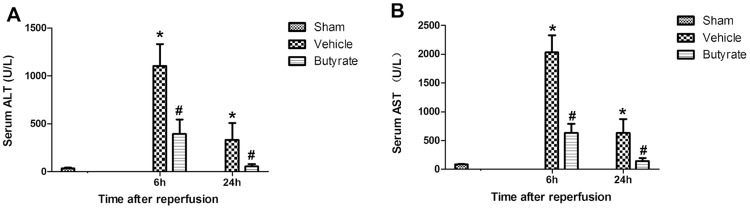
Effect of butyrate on serum ALT and AST levels after reperfusion. Rats were subjected to total warm liver I/R injury or sham operation and pretreated with butyrate or vehicle. Serum ALT (A) and AST (B) levels were analyzed as measures of hepatocellular injury at 6 h and 24 h after reperfusion. Data represent means ± SD, N = 3–5 rats per group. *P<0.05 vs. the sham group, ^#^P<0.05 vs. the vehicle group.

**Figure 2:**
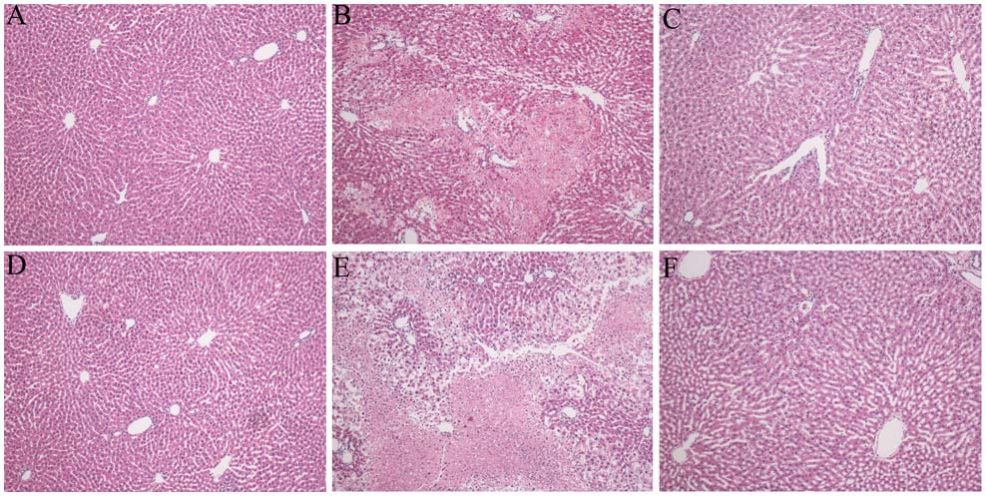
Histopathologic analyses of livers after reperfusion. Rats were subjected to total warm liver I/R injury or sham operation and pretreated with butyrate or vehicle. HE-stained liver sections from the sham (A, D), vehicle (B, E), and butyrate (C, F) groups at 6 h (B, C) and 24 h (E, F) after reperfusion (×200).

Rats pretreated with either vehicle or sodium butyrate were subjected to 30 min of total hepatic I/R. The serum ALT and AST levels significantly increased in the vehicle group at 6 and 24 h after reperfusion (Figure 1). In contrast, pretreatment with butyrate significantly decreased the serum levels of AST and ALT at the observation points. The protection was also confirmed by liver pathology after reperfusion (Figure 2). Severe lobular distortion with massive necrosis, increased swelling, cytoplasmic vacuolization, hemorrhage, and neutrophil infiltration were present in the portal area of liver tissue in the vehicle group. In contrast, pretreatment with butyrate markedly reduced the above pathologic changes; mild damage characterized by interstitial edema and less neutrophil infiltration were observed only in a few areas of the liver in the butyrate group.

### Butyrate decreases liver I/R-induced inflammatory cytokines production

**Figure 3:**
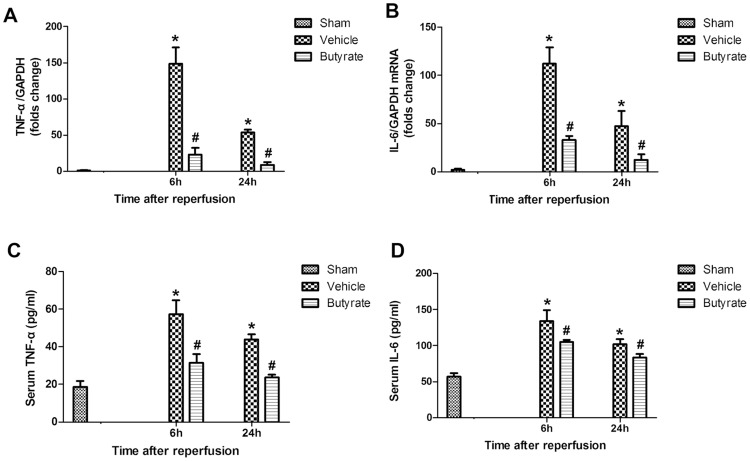
Effect of butyrate on inflammatory cytokines production. Rats were subjected to total warm liver I/R injury or sham operation and pretreated with butyrate or vehicle. Liver TNF-α (A) and IL-6 (B) mRNA expression was measured by RT-PCR after reperfusion. Serum TNF-α (C) and IL-6 (D) levels was measured by Elisa after reperfusion. Data represent means ± SD, N = 3–5 rats per group. *P<0.05 vs. the sham group, ^#^P<0.05 vs. the vehicle group.

Inflammatory cytokines such as TNF-α and IL-6 contribute to the pathophysiology of hepatic I/R injury. We analyzed the rats’ mRNA expression patterns in the liver following I/R using RT-PCR. As shown in Figure 3A and 3B, butyrate significantly decreased the intrahepatic expression of mRNA coding for TNF-α and IL-6 at 6 and 24 h after reperfusion in comparison with the vehicle group. Furthermore, the serum levels of TNF-α and IL-6 assessed by ELISA were consistent with the mRNA results (Figure 3C, D).

### Butyrate inhibits macrophages activation, TLR4 expression in live

**Figure 4:**
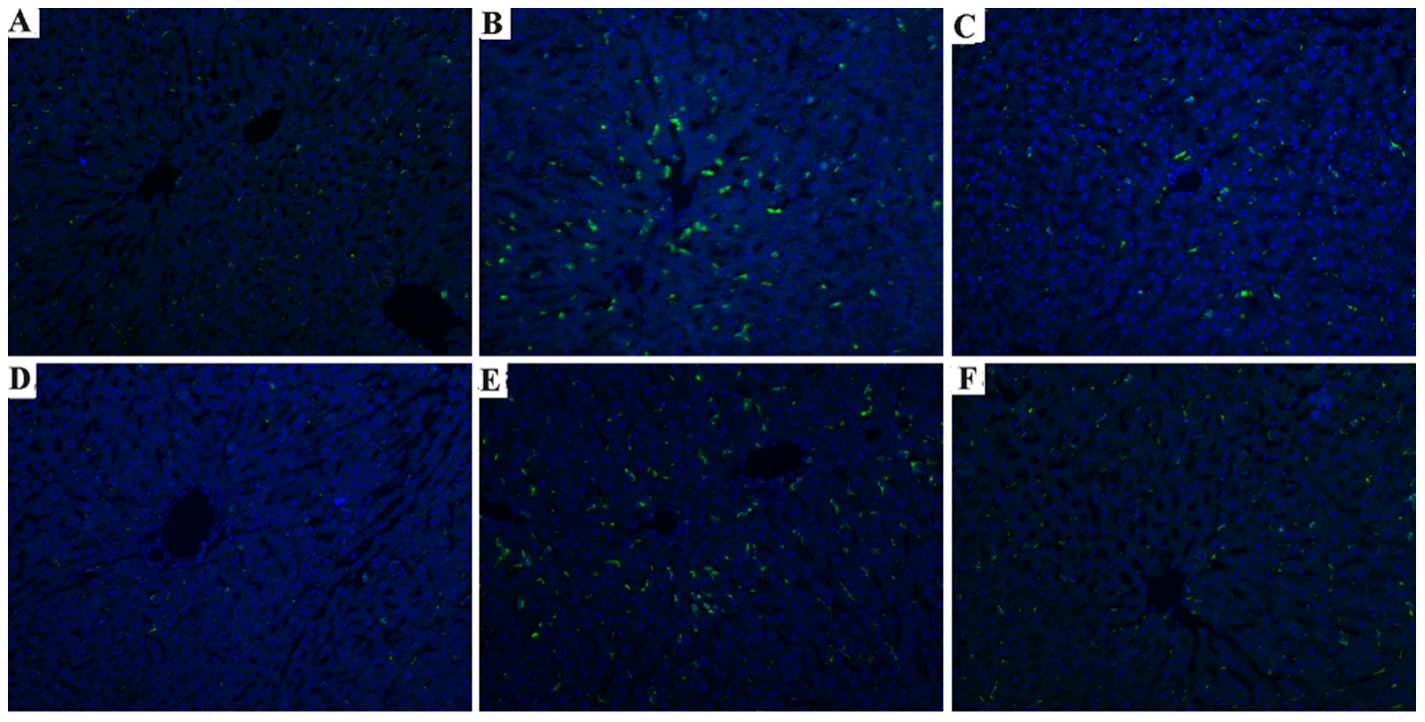
Effect of butyrate on macrophages activation. Rats were subjected to total warm liver I/R injury or sham operation and pretreated with butyrate or vehicle. Immunofluorescent staining of CD68 in the sham (A, D), vehicle (B, E), and butyrate (C, F) groups at 6 h (B, C) and 24 h (E, F) after reperfusion (×200).

**Figure 5:**
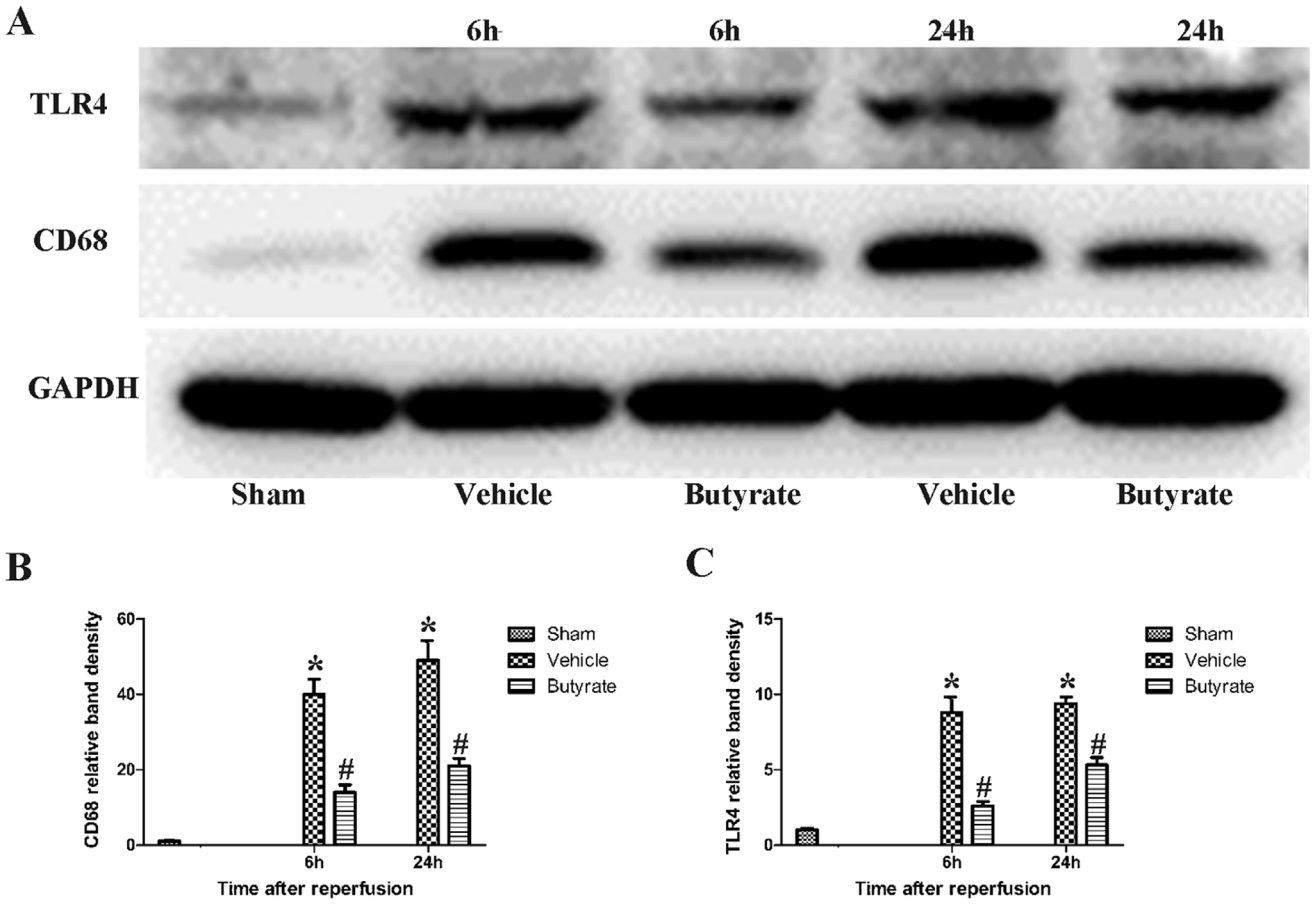
Effect of butyrate on CD68 and TLR4 expression in live. Rats were subjected to total warm liver I/R injury or sham operation and pretreated with butyrate or vehicle. Western blots (A) and quantitative evaluation (B, C) of the expression of CD68 and TLR4 in live after I/R. Data represent means ± SD, N = 3–5 rats per group. *P<0.05 vs. the sham group, ^#^P<0.05 vs. the vehicle group.

Kupffer cells and liver-infiltrating monocyte-derived macrophages play important roles during liver I/R injury [16]. CD68 was detected as the marker of activated macrophages [17]. Immunofluorescent staining showed that only a few CD68-positive cells in the sham group (Figure 4); the CD68-positive cells in the vehicle hepatic tissue increased prominently. However, butyrate treatment significantly inhibited the macrophages activation at 6 and 24 h post-reperfusion (Figure 4). The protein expression assessed by Western blot revealed that the CD68 expression in the liver was upregulated significantly after total hepatic I/R, which was remarkably downregulated compared with the vehicle group by butyrate (Figure 5).

Hepatic I/R injury and LPS signaling are largely TLR4 dependent [16], [18]. The expression of TLR4 was determined by Western blot. As shown in Figure 5., there was a marked increase in the expression of TLR4 after total hepatic I/R, but less so in the butyrate group, indicting the inhibition of inflammation cascade by butyrate.

### Butyrate inhibits neutrophil infiltration in live

**Figure 6:**
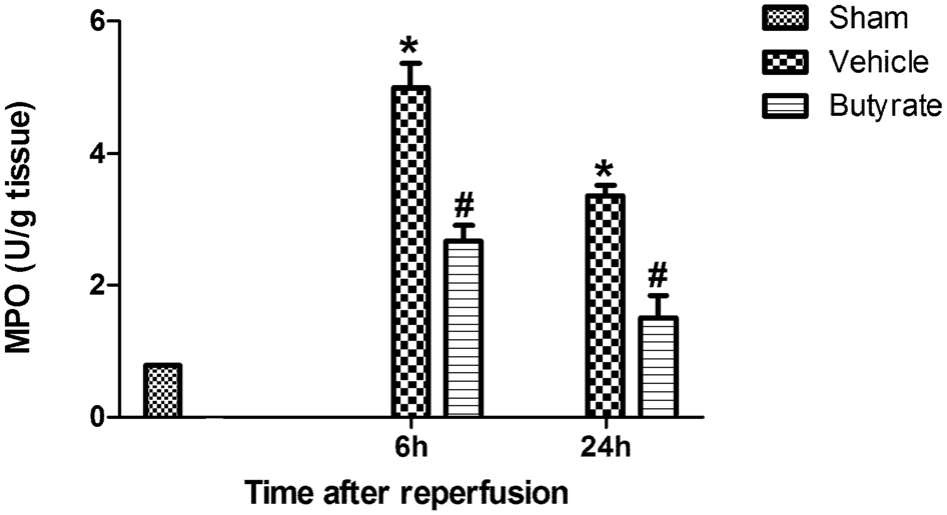
Effect of butyrate on MPO activity. Rats were subjected to total liver I/R injury or sham operation and pretreated with butyrate or vehicle. MPO activity in the liver was measured after reperfusion. Data represent means ± SD, N = 3–5 rats per group. *P<0.05 vs. the sham group, ^#^P<0.05 vs. the vehicle group.

Based on the pivotal mediators of neutrophils in inflammatory response during I/R injury, we also tested the role of neutrophils infiltration in butyrate live protecting. Neutrophil infiltration after reperfusion in the vehicle group, as analyzed by MPO activity (U/g tissue), increased significantly. Compared to the vehicle group at 6 to 24 h after reperfusion, butyrate pretreatment reduced MPO activity (Figure 6), suggesting that butyrate may inhibit neutrophil infiltration.

### Butyrate attenuates serum endotoxin concentration

**Figure 7:**
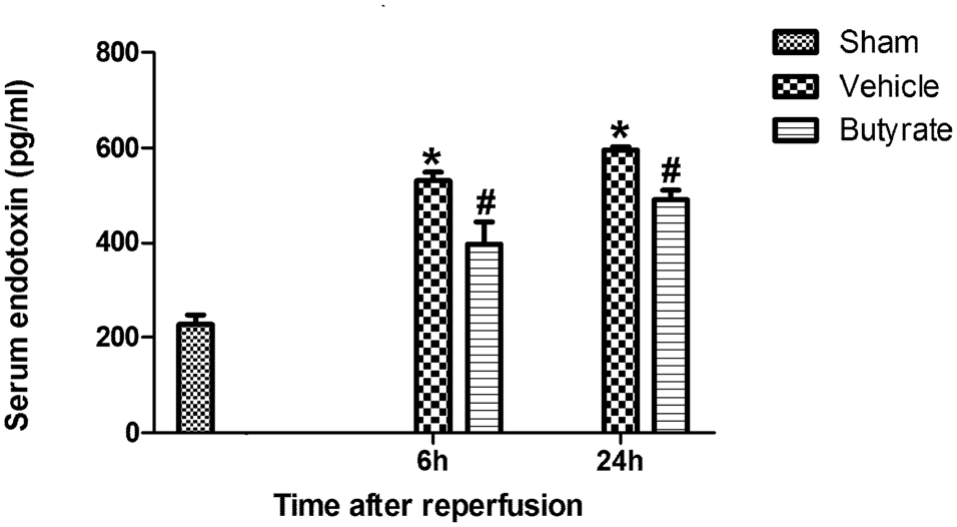
Effect of butyrate on serum endotoxin concentration. Rats were subjected to total liver I/R injury or sham operation and pretreated with butyrate or vehicle. Serum endotoxin concentration was measured by Elisa after reperfusion. Data represent means ± SD, N = 3–5 rats per group. *P<0.05 vs. the sham group, ^#^P<0.05 vs. the vehicle group.

The link between endotoxin and liver injury has been well demonstrated in other studies. In our study, the endotoxin level in the portal vein was found to be remarkably increased after reperfusion (Figure 7); however, it was decreased in the butyrate group.

### Butyrate attenuates intestinal mucosal injury and epithelial apoptosis

**Figure 8:**
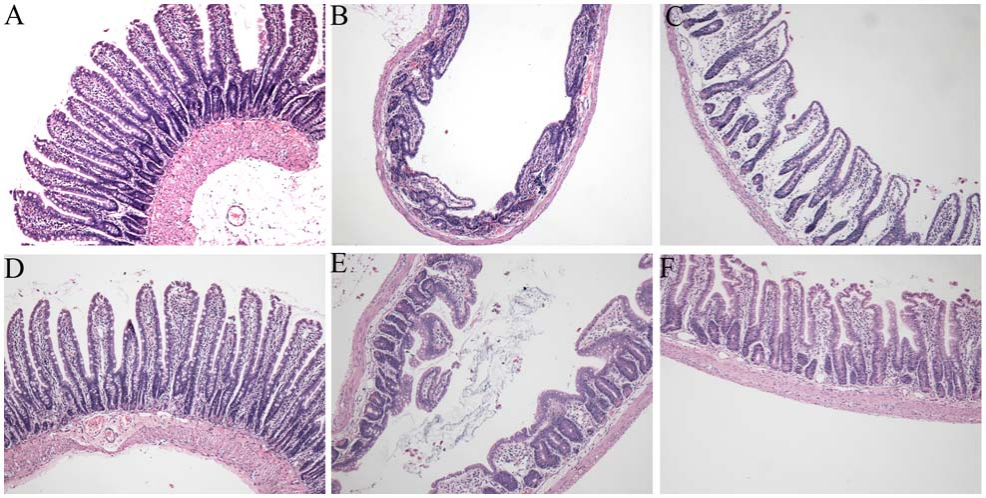
Histopathologic analyses of intestinal mucosa after reperfusion. Rats were subjected to total warm liver I/R injury or sham operation and pretreated with butyrate or vehicle. HE-stained intestinal mucosa from the sham (A, D), vehicle (B, E), and butyrate (C, F) groups at 6 h (B, C) and 24 h (E, F) after reperfusion (×200).

Histopathologic analysis of the control rats showed a normal mucosal pattern with packed, tall, and intact villi (Figure 8A, D). Compared with the control animals, total hepatic I/R insult caused significant mucosal damage, that is, epithelial shedding, villi fracturing, mucosal atrophy, and edema (Figure 8B, E). However, butyrate treatment significantly attenuated the mucosal damage at 6 and 24 h post-reperfusion (Figure 8C, F).

**Figure 9:**
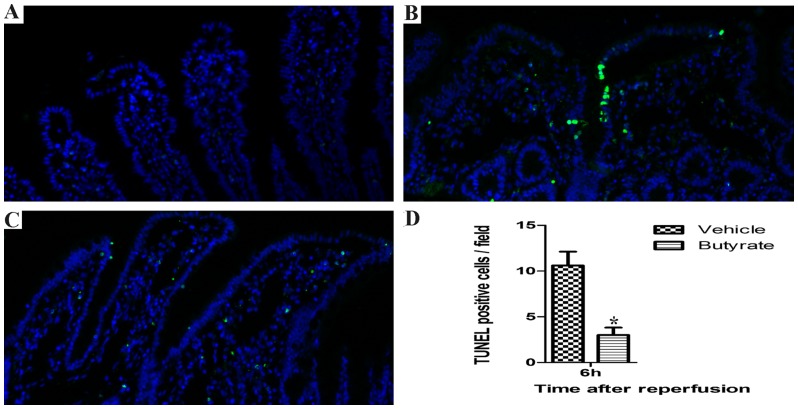
Effects of butyrate on intestinal mucosal epithelial cell apoptosis after I/R. Rats were subjected to total liver I/R injury or sham operation and pretreated with butyrate or vehicle. TUNEL staining was performed to detect intestinal mucosal epithelial cell apoptosis in the sham (A), vehicle (B), and butyrate (C) groups at 6 h after reperfusion (×200). The number of positive cells is presented as means ± SD (D), N = 3–5 rats per group. *P<0.05 vs. the sham group,^ #^P<0.05 vs. the vehicle group.

To further determine the intestinal epithelial barrier integrity after total hepatic I/R, TUNEL staining was performed. After 6 h of reperfusion, the vehicle group demonstrated the highest number of apoptotic cells when compared with the sham group (Figure 9). In contrast, butyrate significantly inhibited the epithelial apoptosis when compared with the vehicle group (Figure 9).

### Butyrate prevents ultrastructure alteration of tight junctions

**Figure 10:**
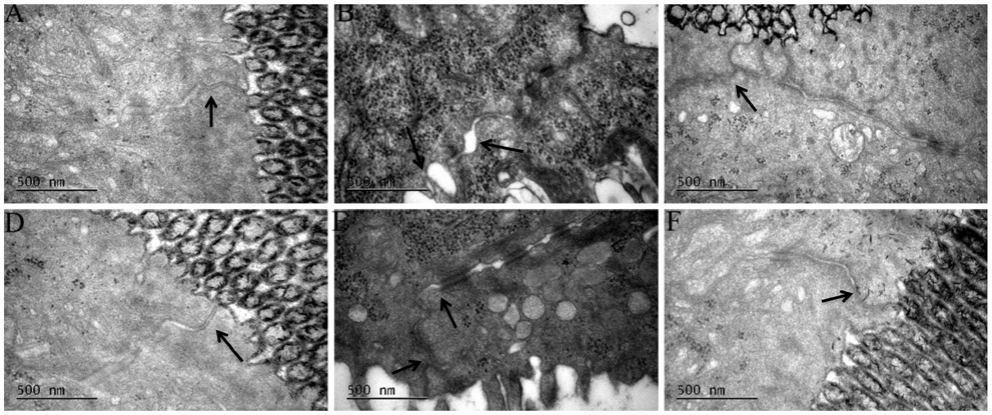
Transmission electron microscopy of intestine. Rats were subjected to total warm liver I/R injury or sham operation and pretreated with butyrate or vehicle. Transmission electron microscopy of rat intestine from the sham (A, D), vehicle (B, E), and butyrate (C, F) groups at 6 h (B, C) and 24 h (E, F) after reperfusion, focusing on tight junctions (↑).

The intestinal ultrastructure was evaluated to analyze the influence of butyrate on tight junctions (TJs). The 30 min ischemia and sequent 6 h reperfusion resulted in obvious ultrastructural changes in the intestinal mucosa, including epithelial sparsely distributing, disarranging, and distorting cell microvilli; epithelial cell edema or shrinkage; dilation of the rough endoplasmic reticulum, mitochondrial swelling and crista fragmentation; and TJs membrane fusion disruption (Figure 10B, E). In contrast, butyrate supplementation alleviated these ultrastructural pathological changes (Figure 10C, F).

### Butyrate protect of the TJs protein ZO-1 after I/R injury

**Figure 11:**
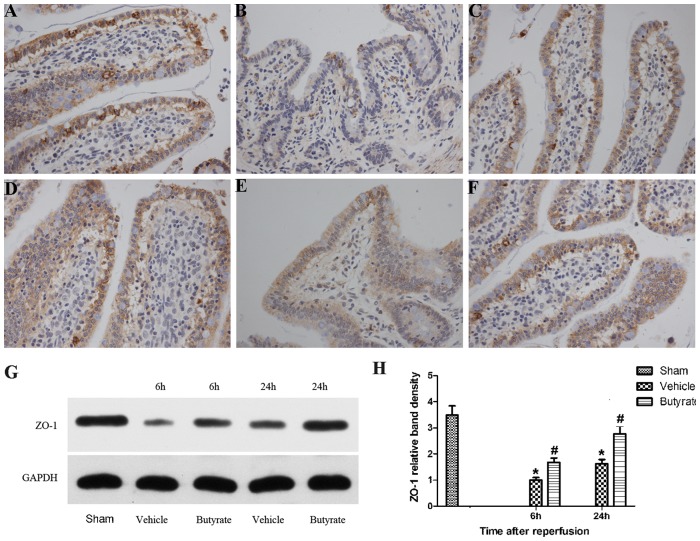
Effects of butyrate on expression of ZO-1. Rats were subjected to total liver I/R injury or sham operation and pretreated with butyrate or vehicle. Immunohistochemical staining of ZO-1 was detected in the sham (A, D), vehicle (B, E), and butyrate (C, F) groups at 6 h (B, C) and 24 h (E, F) after reperfusion (×400). Representative Western blots analysis (G) and quantitative evaluation (H) of the expression of ZO-1 in intestinal mucosa after I/R. GAPDH was run as an internal standard. Data represent means ± SD, N = 3–5 rats per group. *P<0.05 vs. the sham group, ^#^P<0.05 vs. the vehicle group.

TJs proteins play a critical role in the maintenance of mucosal barrier function, whose deficiency is associated with the altered expression and distribution of TJs proteins. Immunohistochemistry demonstrated the expression of TJs protein ZO-1 in linear fashion at the apical surface of the epithelium with a typical reticular pattern in a normal state (Figure 11A, C). After reperfusion, the expression of ZO-1 showed a significantly disrupted, diffuse staining pattern (Figure 11B, E). Western blot analysis confirmed that ZO-1 expression decreased more significantly after reperfusion, particularly at 6 h (Figure 11G, H). However, butyrate improved the aberrant expression of ZO-1 following I/R, and the reticular structures were subtotally maintained (Figure 11C, F). Consistent with the immunohistochemical results, the expression of ZO-1 was reversed by supplementation with butyrate, as shown by Western blot analysis (Figure 11G, H). These results suggest that butyrate can exert a protective effect on the TJs proteins after reperfusion injury with bowel congestion.

## Discussion

The present study demonstrated that I/R resulted in liver injury, as evidenced by the elevated serum levels of AST, ALT, and tissue pathologic changes. Previous research has shown a marked increase in sensitivity to endotoxin after liver injury, leading to further hepatic damage and even systemic endotoxemia or septic shock [19]. Several studies have demonstrated that I/R-induced liver injury was aggravated by exogenous LPS [6],[20],[21]. Thus, decreasing the levels of endotoxin may protect the liver from injury. The results presented here are promising, as pretreatment with butyrate attenuates liver injury induced by I/R aggravated by endotoxin translocated from the intestinal lumen due to dysfunction of the gut barrier function, as indicated by the reduced transaminase levels and improved tissue pathology.

What calls for special attention here is that our model of total hepatic I/R with bowel congestion differs from other models involving partial hepatic ischemia with or without LPS administration. In the setting of liver transplantation, portal vein interrupt and bowel congestion are unavoidable. To better reflect the pathophysiology but simplify the procedure of hepatic transplantation, we employed the total hepatic I/R model in our study.

HDAC inhibition is emerging as a novel approach to treat a variety of diseases. Recently, butyrate has been shown to have anti-inflammatory effects both in vitro and in vivo [22], [23], [24]. HDAC inhibitors have previously shown robust neuroprotective effects in a focal cerebral ischemia model of rats [25], protecting the heart against ischemic injury [26]. Our previous study also supported the pivotal role of butyrate in protection of hepatic injury in the rat partial I/R model [13]. We are also interested in whether butyrate can attenuate total hepatic I/R injury complicated by endotoxin from the intestinal lumen.

Endotoxin is normally prevented from entering the intestinal lumen because of the intestinal barrier. The intestinal barrier function depends on TJs between intact epithelial cells [27], [28]. The disruption of the epithelial barrier because of bowel congestion in total hepatic I/R results in increased intestinal permeability, permitting endotoxin to translocate from the lumen into the portal blood. This, in turn, causes secondary insult to hepatic injury, and even liver failure. Therefore, in the early phase of reperfusion injury, if we can attenuate intestinal barrier injury to inhibit endotoxin translocation, the secondary insult may be reduced.

Few studies have examined intestinal barrier injury and the TJs ultrastructure during total hepatic I/R injury, and butyrate’s role and mechanism of action in total hepatic I/R injury remain unclear. In the present study, we observed gross morphological changes and the ultrastructure of microvilli in intestinal tissue. Obvious morphological changes were found, including the shedding and apoptosis of epithelial cells, fracturing and fusion of villi, mucosal atrophy, and edema. This histological damage indicated intestinal barrier function injury, possibly resulting in increased permeability. Moreover, we indeed observed increased endotoxin levels after reperfusion, with simultaneous disruption to TJs integrity. Additionally, these pathological changes were mitigated with butyrate administration, and the endotoxin levels were reduced. Therefore, the increase in intestinal permeability may be attributed to the disruption of TJs between intestinal mucosal epithelial cells, whereas butyrate reversed this pathological change.

To analyze the mechanism of TJs disruption, we assessed the expression of the TJs-related protein ZO-1 by immunohistochemistry and western blot. ZO-1 has been demonstrated to interact between transmembrane protein occlusion and the actin cytoskeleton and play a crucial role in the maintenance of the integrity of the intestinal mucosal barrier and TJs in numerous pathological and physiological processes [29]. In this study, total hepatic I/R injury resulted in the abnormal distribution of ZO-1 and significantly decreased ZO-1 expression in the intestinal mucosa, whereas butyrate pretreatment led to a reversal of aberrant ZO-1 expression, which was probably associated with the mechanisms by which butyrate inhibits gut leakage induced by bowel congestion in total hepatic I/R.

KCs, the resident macrophages in the liver, have been identified as the primary cell type in the initiation and perpetuation of I/R injury [16], which can be further activated by endotoxin through TLR4. Indeed, activated KCs produce reactive oxygen species, proinflammatory cytokines and chemokines. These recruit and activate circulating macrophages, neutrophils, lymphocytes, and sinusoidal endothelial cells, all of which contribute to the inflammation associated with liver damage [1]. We found that CD68, the marker of peripheral or liver-resident macrophages (KCs), is increased in the liver tissue after reperfusion, suggesting that total I/R had induced more macrophages activation, which was confirmed by the remarkable heightened TNF-α and IL-6 levels. Meanwhile, MPO activity, which is a widely used marker of neutrophil infiltration, was rapidly upregulated. However, pre-administration of butyrate downregulated the expression of CD68 protein and MPO activity, decreased inflammatory cytokines, which obviously indicates inhibition of KC activation and neutrophil infiltration, thereby ameliorating the liver injury.

Increasing evidence suggests that TLR4, the detector of bacterial endotoxin and other endogenous ligands including high-mobility group box 1, plays a critical role in LPS signaling and pathogenesis of liver I/R injury [16]. We further detected the increased expression of TLR4 and downregulation by butyrate administration, confirming the hepatoprotection of butyrate.

In conclusion, our study suggests that butyrate hepatoprotection in a rat model of total hepatic I/R injury may be associated with decreased endotoxin translocation via protection of gut barrier function injury and suppressing macrophages activation, inflammatory factor production and neutrophil infiltration. Given that butyrate is a naturally occurring product in the body with low toxicity, it may be an effective hepatoprotective agent and a promising candidate in liver transplantation.
